# Supplementary material for: Transforming a Large-Scale Prostate Cancer Outcomes Dataset to the OMOP Common Data Model—Experiences from a Scientific Data Holder’s Perspective
Source: Cancers (Basel). 2024 May 30;16(11):2069. doi: 10.3390/cancers16112069 (PMC11171220; doi:10.3390/cancers16112069)
Supplement: Supplementary file 1 [file cancers-16-02069-s001.zip › cancers-3013748-supplementary.pdf]

Supplementary S1: Most frequently mapped concepts

Table S1. Top 25 of mapped **conditions**. Counts are rounded up to the nearest hundred.

#	Concept Name	#Records	#Subjects
1	Regular bowel action	63,300	43,100
2	Normal bowel habits	63,000	43,100
3	Decreased sexual function	57,500	40,100
4	Normal sexual function	48,400	38,600
5	Bladder: fully continent	46,200	36,100
6	Urgent desire to urinate	43,200	32,400
7	Normal urinary stream	41,200	31,100
8	Lack of energy	35,100	26,200
9	Poor stream of urine	34,600	28,600
10	Residual tumor stage R0	34,000	34,000
11	Altered bladder function	33,400	26,300
12	Intermittent urinary incontinence	31,000	25,000
13	Dribbling of urine	30,600	24,500
14	Depressed mood	28,900	22,200
15	Able to have sexual intercourse	26,000	23,600
16	Problem getting an erection	25,700	21,600
17	Cannot get an erection	24,000	19,700
18	Poor erection	24,000	17,100
19	Orgasm incapacity	19,900	16,500
20	Change in body weight	19,100	16,000
21	Irregular bowel habits	15,500	12,900
22	Erection firm enough for masturbation and foreplay only	15,300	13,900
23	Urgent desire for stool	13,900	11,700
24	Lack or loss of sexual desire	13,800	11,000
25	Abdominal/Pelvic/Rectal pain	13,500	11,400

Query executed in 1.23 secs

Table S2. Top 25 of mapped **measurements**. Counts are rounded up to the nearest hundred.

#	Concept Name	#Records	#Subjects
1	PSA (prostate-specific antigen) level	71,700	49,300
2	Gleason Primary Pattern Grade 3	54,800	33,700
3	Number of Cores Examined	49,300	49,300
4	AJCC/UICC clinical M0 Category	49,300	49,300
5	Gleason Secondary Pattern Grade 4	49,300	34,400
6	Number of Cores Positive	49,300	49,300
7	AJCC/UICC clinical N0 Category	48,700	48,700
8	AJCC/UICC pathological M0 Category	42,900	42,900
9	Greatest percentage of prostate core involved by tumor	42,200	42,200
10	AJCC/UICC pathological N0 Category	37,400	37,400
11	Surgery regional nodes examined [#] Cancer	36,000	36,000
12	Number of regional lymph nodes involved	35,900	35,900
13	Gleason Secondary Pattern Grade 3	35,700	27,200
14	Gleason Primary Pattern Grade 4	35,200	23,500
15	Source of follow-up information Cancer	34,300	34,300
16	AJCC/UICC clinical T1c Category	32,600	32,600
17	AJCC/UICC pathological T2c Category	23,700	23,700
18	AJCC/UICC pathological T3a Category	9,400	9,400

#	Concept Name	#Records	#Subjects
19	Total radiation dose delivered	7,600	7,600
20	Gleason Secondary Pattern Grade 5	7,500	5,800
21	AJCC/UICC pathological T3b Category	6,300	6,300
22	Extent of Margin Involvement Focal	5,900	5,900
23	AJCC/UICC clinical T2c Category	5,200	5,200
24	AJCC/UICC clinical T2a Category	5,200	5,200
25	AJCC/UICC pathological N1 Category	4,100	4,100

Query executed in 1.23 secs

Table S3. Top 25 of mapped **observations**. Counts are rounded up to the nearest hundred.

#	Concept Name	#Records	#Subjects
1	Assessment score	374,600	49,200
2	Condition severity	355,100	46,700
3	Prostate Cancer Outcomes (PCO) questionnaire	79,300	49,300
4	Sexual function	77,100	48,600
5	Erection frequency	76,500	48,300
6	Care and treatment plan review meeting	73,000	48,800
7	Erotic interest	64,100	43,000
8	Ability to reach orgasm	56,000	41,100
9	Health insurance	45,600	45,600
10	Educational achievement	44,800	44,800
11	Country of citizenship	43,000	43,000
12	Ability to get an erection	40,000	33,900
13	Follow-up status	39,500	39,500
14	Under follow-up	34,500	34,500
15	Disease Episode	34,200	34,200
16	Frequency of behaviour	20,400	17,500
17	Comorbidities and coexisting conditions	17,700	13,500
18	Use of	14,200	13,300
19	Average fraction of radiation dose	7,600	7,600
20	Device used	2,800	2,800
21	Active surveillance	2,100	2,100
22	Current use of device	2,000	2,000
23	Complications	1,800	1,300
24	History of event	1,400	1,400
25	Past medication	1,100	1,000

Query executed in 2.07 secs

Table S4. All 24 mapped **procedures**. Counts are rounded up to the nearest hundred.

#	Concept Name	#Records	#Subjects
1	Core needle biopsy of prostate	49,300	49,300
2	Radical excision of lymph nodes	38,100	38,100
3	Social service interview of patient	37,000	37,000
4	Nerve-sparing surgery	29,100	29,100
5	Robot assisted laparoscopic radical prostatectomy	23,200	23,200
6	Psychological counseling	17,000	17,000
7	Radical retropubic prostatectomy	11,400	11,400
8	External beam radiation therapy procedure	7,600	7,600
9	Radical prostatectomy	5,300	5,300
10	Androgen deprivation therapy	4,200	3,900
11	Laparoscopic radical prostatectomy	3,100	3,100

#	Concept Name	#Records	#Subjects
12	Revision	1,300	1,300
13	Low dose rate brachytherapy	400	400
14	Patient status observation	300	300
15	Local therapy	200	200
16	Radical perineal prostatectomy	200	200
17	High dose rate electronic brachytherapy	200	200
18	High intensity focused ultrasound of prostate	100	100
19	Drug therapy	100	100
20	Chemotherapy	100	100
21	Radical cystoprostatectomy	100	100
22	Robot assisted laparoscopic radical cystectomy	100	100
23	Immunological therapy	100	100
24	Neoadjuvant chemotherapy	100	100

Supplementary S2: Overview of all used vocabularies

Vocabulary version: v5.0 31-MAY-23

The vocabularies available in the CDM with concept count. Note that this does not reflect which concepts are actually used in the clinical CDM tables. S=Standard, C=Classification and '-'=Non-standard

ID	NAME	VERSION	S	C	-
ABMS	Provider Specialty (American Board of Medical Specialties)	2018-06-26 ABMS	85	0	13
AMT	Australian Medicines Terminology (NEHTA)	Clinical Terminology v20210630	6,839	0	130,011
APC	Ambulatory Payment Classification (CMS)	2018-January-Addendum-A	715	0	1,195
ATC	WHO Anatomic Therapeutic Chemical Classification	RxNorm 20210907	0	6,509	231
BDPM	Public Database of Medications (Social-Sante)	BDPM 20191006	1,106	0	43,270
CCAM	Common Classification of Medical Acts (ATIH)	CCAM version 64	0	0	10,206
CDM	OMOP Common DataModel	CDM v6.0.0	1,060	0	0
CGI	Cancer Genome Interpreter (Pompeu Fabra University)	CGI20180216	0	0	5,351
CIEL	Columbia International eHealth Laboratory (Columbia University)	Openmrs 1.11.0 20150227	0	0	50,881
CIM10	International Classification of Diseases, Tenth Revision, French Edition (ATIH)	CIM10 2022	0	0	12,226
CIViC	Clinical Interpretation of Variants in Cancer (civicdb.org)	CIViC 2022-10-01	0	0	1,386
CMS Place of Service	Place of Service Codes for Professional Claims (CMS)	2009-01-11	51	0	9
CTD	Comparative Toxicogenomic Database (NCSU)	CTD 2020-02-19	0	0	8,698
CVX	CDC Vaccine Administered CVX (NCIRD)	CVX 20230418	223	0	33
Cancer Modifier	Diagnostic Modifiers of Cancer (OMOP)	Cancer Modifier 20220909	5,317	38	688

ID	NAME	VERSION	S	C	-
ClinVar	ClinVar (NCBI)	ClinVar v20200901	0	0	8,072
Cohort	Legacy OMOP HOI or DOI cohort	NA	0	78	0
Cohort Type	OMOP Cohort Type	NA	0	0	1
Concept Class	OMOP Concept Class	NA	0	0	417
Condition Status	OMOP Condition Status	NA	22	0	0
Condition Type	OMOP Condition Occurrence Type	NA	0	0	118
Cost	OMOP Cost	NA	51	0	0
Cost Type	OMOP Cost Type	NA	0	0	8
Currency	International Currency Symbol (ISO 4217)	2008	180	0	0
DKG_OMOP	DKG_OMOP	version 15-10-2023	165	0	0
DPD	Drug Product Database (Health Canada)	DPD 25-JUN-17	0	0	193,647
DRG	Diagnosis-related group (CMS)	2011-18-02	752	0	610
Death Type	OMOP Death Type	NA	0	0	14
Device Type	OMOP Device Type	NA	0	0	4
Domain	OMOP Domain	NA	0	0	65
Drug Type	OMOP Drug Exposure Type	NA	0	0	16
EDI	Korean Electronic Data Interchange code system (HIRA)	EDI 2019.10.01	0	0	313,431
EphMRA ATC	Anatomical Classification of Pharmaceutical Products (EphMRA)	EphMRA ATC 2016	0	895	0
Episode	OMOP Episode	Episode 20201014	14	0	4
Episode Type	OMOP Episode Type	NA	0	0	5
Ethnicity	OMOP Ethnicity	NA	2	0	0
GCN_SEQNO	Clinical Formulation ID (FDB)	20151119 Release	0	0	29,659
GGR	Commented Drug Directory (BCFI)	GGR 20210901	751	0	26,457
Gender	OMOP Gender	NA	2	0	3
HCPCS	Healthcare Common Procedure Coding System (CMS)	20230701 Alpha Numeric HCPCS File	7,741	0	3,816
HES Specialty	Hospital Episode Statistics Specialty (NHS)	2018-06-26 HES Specialty	57	0	108
HemOnc	HemOnc	HemOnc 2022-11-29	2,185	378	5,465
ICD10	International Classification of Diseases, Tenth Revision (WHO)	2021 Release	0	0	16,519
ICD10CM	International Classification of Diseases, Tenth Revision, Clinical Modification (NCHS)	ICD10CM FY2023 code descriptions	0	0	98,583
ICD10CN	International Classification of Diseases, Tenth Revision, Chinese Edition (CAMS)	2016 Release	0	0	34,491
ICD10GM	International Classification of Diseases, Tenth Revision, German Edition	ICD10GM 2022	0	0	17,213
ICD10PCS	ICD-10 Procedure Coding System (CMS)	ICD10PCS 2021	194,874	0	107
ICD9CM	International Classification of Diseases, Ninth Revision, Clinical	test	0	0	17,564

ID	NAME	VERSION	S	C	-
	Modification, Volume 1 and 2 (NCHS)				
ICD9Proc	International Classification of Diseases, Ninth Revision, Clinical Modification, Volume 3 (NCHS)	ICD9CM v32 master descriptions	2,223	0	2,434
ICD9ProcCN	International Classification of Diseases, Ninth Revision, Chinese Edition, Procedures (CAMS)	2017 Release	0	0	13,385
ICDO3	International Classification of Diseases for Oncology, Third Edition (WHO)	ICDO3 SEER Site/Histology Released 06/2020	56,972	0	7,499
JAX	The Clinical Knowledgebase (The Jackson Laboratory)	JAX v20200824	0	0	7,855
JMDC	Japan Medical Data Center Drug Code (JMDC)	JMDC 2020-04-30	1,313	0	37,485
KCD7	Korean Standard Classfication of Diseases and Causes of Death, 7th Revision (STATISTICS KOREA)	7th revision	0	0	22,508
KDC	Korean Drug Code (HIRA)	KDC 2020-07-31	112	0	63,749
KNHIS	Korean Payer (KNHIS)	NA	3	0	0
Korean Revenue Code	Korean Revenue Code (KNHIS)	NA	7	0	0
LOINC	Logical Observation Identifiers Names and Codes (Regenstrief Institute)	LOINC 2.73	113,893	49,168	102,015
Language	OMOP Language	Language 20221030	1	0	0
MDC	Major Diagnostic Categories (CMS)	2013-01-06	26	0	0
MMI	Modernizing Medicine (MMI)	NA	4	0	0
MeSH	Medical Subject Headings (NLM)	2023 Release	0	0	352,735
Meas Type	OMOP Measurement Type	NA	0	0	12
Medicare Specialty	Medicare provider/supplier specialty codes (CMS)	2018-06-26 Specialty	112	0	8
Metadata	OMOP Metadata	NA	1	0	1
Multum	Cerner Multum (Cerner)	2013-07-10	0	0	9,770
NAACCR	Data Standards & Data Dictionary Volume II (NAACCR)	NAACCR v18	22,807	0	11,666
NCCD	Normalized Chinese Clinical Drug knowledge base (UTHealth)	NCCD_v02_2020	0	0	51,583
NCIt	NCI Thesaurus (National Cancer Institute)	NCIt 20220509	0	0	2,426
NDC	National Drug Code (FDA and manufacturers)	NDC 20230514	11,427	0	1,145,035
NDFRT	National Drug File - Reference Terminology (VA)	RXNORM 2018-08-12	0	0	69,567
NFC	New Form Code (EphMRA)	NFC 20160704	0	692	0
NUCC	National Uniform Claim Committee Health Care Provider Taxonomy Code Set (NUCC)	2018-06-26 NUCC	674	0	181
Nebraska Lexicon	Nebraska Lexicon (UNMC)	Nebraska Lexicon 20190816	4,187	0	461,614
None	OMOP Standardized Vocabularies	v5.0 31-MAY-23	0	0	1

ID	NAME	VERSION	S	C	-
Note Type	OMOP Note Type	NA	0	0	10
OMOP Extension	OMOP Extension (OHDSI)	OMOP Extension 20230531	1,219	0	53
OMOP Genomic	OMOP Genomic vocabulary	OMOP Genomic 20210727	79,791	0	41,200
OMOP Invest Drug	OMOP Investigational Drugs	OMOP Invest Drug version 2022-05-12	0	0	29,727
OPCS4	OPCS Classification of Interventions and Procedures version 4 (NHS)	2021 Release	2,373	0	8,627
OPS	Operations and Procedures Classification (OPS)	OPS Version 2022	0	0	42,959
OSM	OpenStreetMap (OSMF)	OSM Release 2019-02-21	203,339	0	0
OXMIS	Oxford Medical Information System (OCHP)	NA	0	0	8,118
Obs Period Type	OMOP Observation Period Type	NA	0	0	6
Observation Type	OMOP Observation Type	NA	0	0	29
OncoKB	Oncology Knowledge Base (MSK)	OncoKB v20210502	0	0	5,569
OncoTree	OncoTree (MSK)	OncoTree version 2021-11-02	0	0	885
PCORNet	National Patient-Centered Clinical Research Network (PCORI)	NA	2	0	79
PPI	AllOfUs_PPI (Columbia)	Codebook Version 0.4.43 + COVID + MHWB + SDOH + PFH	2,275	0	4,254
Plan	OMOP Health Plan	NA	11	0	0
Plan Stop Reason	OMOP Plan Stop Reason	NA	13	0	0
Procedure Type	OMOP Procedure Occurrence Type	NA	0	0	97
Provider	OMOP Provider	NA	6	0	0
Race	Race and Ethnicity Code Set (USBC)	Version 1.0	50	0	3
Read	NHS UK Read Codes Version 2 (HSCIC)	NHS READV2 21.0.0 20160401000001 + DATAMIGRATION_25.0.0_20180403000001	0	0	108,945
Relationship	OMOP Relationship	NA	14	0	698
Revenue Code	UB04/CMS1450 Revenue Codes (CMS)	2010 Release	538	0	0
RxNorm	RxNorm (NLM)	RxNorm 20230501	150,793	35,463	119,708
RxNorm Extension	OMOP RxNorm Extension	RxNorm Extension 2023-05-31	1,866,855	0	276,255
SMQ	Standardised MedDRA Queries (MSSO)	Version 14.0	0	318	6
SNOMED	Systematic Nomenclature of Medicine - Clinical Terms (IHTSDO)	2021-07-31 SNOMED CT International Edition; 2021-09-01 SNOMED CT US Edition; 2021-11-24	538,088	0	516,847

ID	NAME	VERSION	S	C	-
		SNOMED CT UK Edition			
SNOMED Veterinary	SNOMED Veterinary Extension (VTSL)	SNOMED Veterinary 20190401	31,994	0	1,690
SOPT	Source of Payment Typology (PHDSC)	SOPT Version 9.2	162	0	6
SPL	Structured Product Labeling (FDA)	NDC 20230514	0	646,651	14,969
Specimen Type	OMOP Specimen Type	NA	0	0	1
Sponsor	OMOP Sponsor	NA	6	0	0
Supplier	OMOP Supplier	NA	0	0	1
Type Concept	OMOP Type Concept	Type Concept 20221030	80	0	0
UB04 Point of Origin	UB04 Claim Source Inpatient Admission Code (CMS)	NA	0	0	23
UB04 Pri Typ of Adm	UB04 Claim Inpatient Admission Type Code (CMS)	NA	6	0	0
UB04 Pt dis status	UB04 Patient Discharge Status Code (CMS)	NA	0	0	55
UB04 Typ bill	UB04 Type of Bill - Institutional (USHIK)	NA	4	0	294
UCUM	Unified Code for Units of Measure (Regenstrief Institute)	Version 1.8.2	1,029	0	89
UK Biobank	UK Biobank (UK Biobank)	version 2020-10-15	3,837	292	15,208
US Census	Census regions of the United States (USCB)	US Census 2017 Release	13	0	0
VA Class	VA National Drug File Class (VA)	RxNorm 20211101	0	0	576
VANDF	Veterans Health Administration National Drug File (VA))	RxNorm 20211004	0	0	40,136
Visit	OMOP Visit	Visit 20211216	19	0	0
Visit Type	OMOP Visit Type	NA	0	0	18
Vocabulary	OMOP Vocabulary	NA	0	0	146
dm+d	Dictionary of Medicines and Devices (NHS)	DMD 2023-05-22	26,607	0	378,824
